# Therapeutic Potential and Mechanisms of Mesenchymal Stem Cells in Coronary Artery Disease: Narrative Review

**DOI:** 10.3390/ijms26115414

**Published:** 2025-06-05

**Authors:** Tejas Patel, Jana Mešić, Shai Meretzki, Tomer Bronshtein, Petar Brlek, Vered Kivity, Samir B. Pancholy, Matko Petrović, Dragan Primorac

**Affiliations:** 1 Apex Heart Institute, Ahmedabad 380059, India; 2 St. Catherine Specialty Hospital, 10000 Zagreb, Croatia; 3 Bonus BioGroup, Haifa 31905, Israel; 4 School of Medicine, Josip Juraj Strossmayer University of Osijek, 31000 Osijek, Croatia; 5 Faculty of Science, Department of Molecular Biology, University of Zagreb, 10000 Zagreb, Croatia; 6 Geisinger Commonwealth School of Medicine, Scranton, PA 18509, USA; 7 School of Medicine, University of Split, 21000 Split, Croatia; 8 Eberly College of Science, The Pennsylvania State University, State College, University Park, PA 16802, USA; 9 Henry C. Lee College of Criminal Justice and Forensic Sciences, University of New Haven, New Haven, CT 06516, USA; 10 Regiomed Kliniken, 96450 Coburg, Germany; 11 School of Medicine, University of Rijeka, 51000 Rijeka, Croatia; 12 Faculty of Dental Medicine and Health, Josip Juraj Strossmayer University of Osijek, 31000 Osijek, Croatia; 13 National Forensic Sciences University, Gandhinagar 382007, India

**Keywords:** mesenchymal stem cell, coronary artery disease, regenerative therapy, precision medicine

## Abstract

Coronary artery disease (CAD) remains a leading cause of global morbidity and mortality despite advances in medical and interventional therapies. Mesenchymal stem cell (MSC) therapy has emerged as a promising regenerative approach for patients with refractory or non-revascularizable CAD. MSCs exhibit unique immunomodulatory, pro-angiogenic, and anti-fibrotic properties, primarily through paracrine mechanisms involving the secretion of cytokines, growth factors, and exosomal microRNAs. Clinical and preclinical studies have demonstrated improvements in myocardial perfusion, left ventricular ejection fraction (LVEF), and functional capacity following MSC-based interventions, particularly in patients with low baseline LVEF and heightened inflammation. Various MSC sources—including bone marrow, adipose tissue, and umbilical cord—offer distinct advantages, while delivery strategies such as intracoronary, intramyocardial, intravenous, and subcutaneous administration impact cell retention and efficacy. Advances in genetic modification, hypoxic preconditioning, and exosome-based therapies aim to enhance MSC survival and therapeutic potency. However, challenges persist regarding cell engraftment, cryopreservation effects, and inter-patient variability. Moving toward precision cell therapy, future approaches may involve stratifying patients by inflammatory status, ischemic burden, and comorbidities to optimize treatment outcomes. MSCs may not yet replace conventional therapies but are increasingly positioned to complement them within a personalized, regenerative framework for CAD management.

## 1. Introduction

Coronary artery disease (CAD) is one of the leading cardiovascular diseases and remains a primary cause of mortality in both developed and developing countries [1,2]. CAD is a chronic inflammatory condition characterized by atherosclerotic changes in the coronary arteries, leading to the narrowing or occlusion of blood vessels and subsequent myocardial ischemia [3]. Previously considered a mere disorder of lipid accumulation, atherosclerosis is now understood as a complex interplay of lipid metabolism, endothelial dysfunction, and immune-mediated inflammatory processes [1]. Recent advancements in understanding CAD pathophysiology emphasize the role of endothelial dysfunction, oxidative stress, and systemic inflammation in plaque formation and progression [2,3]. Genome-wide association studies (GWAS) have also identified genetic risk factors, including variations in chromosome 9p21.3, which have been strongly associated with the premature onset of CAD (1). In addition to genetic predisposition, environmental and lifestyle factors such as smoking, hypertension, diabetes, dyslipidemia, and obesity contribute significantly to disease prevalence. Despite significant advancements in prevention and treatment, CAD remains a major public health concern.

Current therapeutic strategies for CAD include pharmacological interventions such as antiplatelet agents, statins, β-blockers, and calcium channel blockers, alongside invasive procedures like percutaneous coronary intervention (PCI) and coronary artery bypass grafting (CABG) [1,4]. Although PCI and CABG are standard revascularization strategies, a significant subset of patients is classified as having non-revascularized CAD, either due to diffuse, small-vessel disease, extensive comorbidities, or prior failed revascularization attempts. These patients often experience persistent angina, ischemic cardiomyopathy, and worsening heart failure despite optimal medical therapy.

For patients suffering from non-revascularized CAD, novel regenerative approaches such as mesenchymal stem cell (MSC) therapy offer a potential solution by addressing underlying myocardial dysfunction, microvascular insufficiency, and chronic inflammation rather than focusing solely on mechanical restoration of blood flow. MSCs have emerged as a promising therapeutic option due to their immunomodulatory and regenerative pro-angiogenic and anti-fibrotic properties, which they exert primarily through paracrine signaling rather than direct differentiation into cardiomyocytes [3,5,6,7,8]. These cells secrete a range of bioactive molecules, including vascular endothelial growth factor (VEGF), fibroblast growth factor-2 (FGF-2), hepatocyte growth factor (HGF), and exosomal microRNAs, all of which contribute to endothelial repair, inhibition of cardiomyocyte apoptosis, and the reduction in myocardial fibrosis [5,9].

Several MSC sources have been explored for CAD therapy, including bone-marrow-derived MSCs (BM-MSCs), adipose-derived MSCs (AD-MSCs), and umbilical-cord-derived MSCs (UC-MSCs). In addition to MSCs, CD34^+^ endothelial progenitor cells (EPCs) have been investigated for their potential to restore endothelial integrity and induce neovascularization, particularly in patients with diffuse non-revascularizable CAD who exhibit significant microvascular dysfunction [5,8,9,10,11]. This review will comprehensively examine the role of MSC-based therapies in both revascularized and non-revascularized CAD, focusing on clinical indications, MSC sources, dosage levels, delivery methods, and primary and secondary endpoints to provide a detailed assessment of their regenerative potential in cardiovascular medicine.

## 2. Coronary Artery Disease

CAD is a chronic and progressive cardiovascular disorder characterized by the development of atherosclerotic plaques within the coronary arteries, leading to luminal narrowing and impaired myocardial perfusion. It is the leading cause of morbidity and mortality worldwide, contributing to myocardial ischemia, infarction, and heart failure. In 2015, CAD accounted for approximately 9 million deaths [12]. Survivors of myocardial infarction face an increased risk of recurrent events, with a five- to six-fold higher annual mortality rate compared to individuals without CAD [2,12,13].

**Figure 1:**
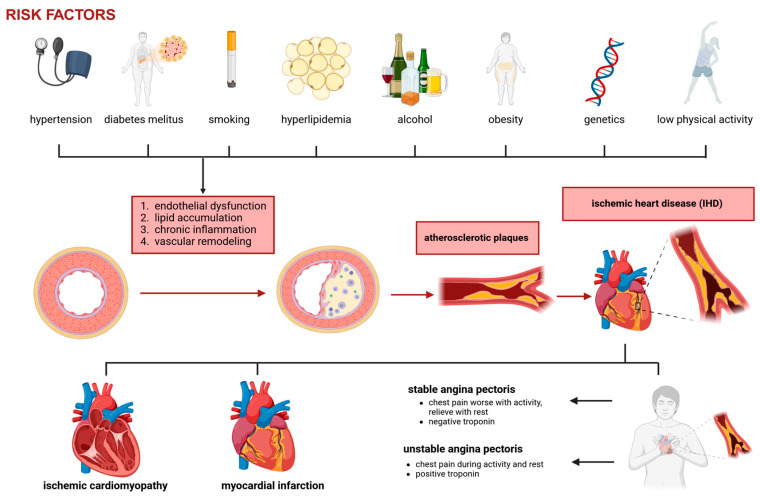
Pathophysiology of coronary artery disease and ischemic heart disease.

While CAD refers to the structural pathology of coronary arteries, ischemic heart disease (IHD) describes the functional consequences arising from impaired myocardial blood flow [14]. In other words, CAD is the underlying disease process, while IHD encompasses the spectrum of clinical manifestations that result from insufficient oxygen delivery to myocardial tissue [15]. The development of CAD is a multifactorial and complex process involving endothelial dysfunction, lipid accumulation, chronic inflammation, and vascular remodeling. It is influenced by both genetic and environmental factors, including hyperlipidemia, hypertension, diabetes mellitus, smoking, and chronic inflammation (Figure 1) [16]. The vascular endothelium is a critical regulator of vascular tone, hemostasis, and immune response.

In the early stages of CAD, endothelial cells lost their ability to maintain vasodilation and anti-inflammatory properties. This dysfunction is driven by oxidative stress, dyslipidemia, and pro-inflammatory cytokines, leading to reduced endothelial nitric oxide synthase (eNOS)–derived nitric oxide (NO) bioavailability, which impairs vasodilation and promotes pro-atherogenic changes in the arterial wall [14]. It is important to distinguish this reduction in entohelial NO from increased NO production by inducible nitric oxide synthase (iNOS) in activated inflammatory cells, which occurs in a different cellular context. Endothelial activation results in the upregulation of adhesion molecules such as VCAM-1 and ICAM-1, which facilitate monocyte adhesion and infiltration into the subendothelial space. These monocytes differentiate into macrophages, which engulf oxidized low-density lipoprotein (oxLDL) and transform into foam cells, forming the fatty streak, the earliest visible stage of atherosclerosis [15]. Once foam cells accumulate, they secrete pro-inflammatory cytokines, including TNF-α, IL-6, and IL-1β, which sustain chronic inflammation and further amplify immune cell infiltration into the plaque [3,7,14,17,18]. The Wnt/β-catenin signaling pathway is suppressed during ischemia-reperfusion injury, leading to increased apoptosis and inflammation, while non-canonical Wnt signaling contributes to calcium overload and oxidative stress. Notch signaling exerts protective effects by interacting with Wnt, reducing infarct size, and promoting myocardial repair [18]. The PI3K/Akt pathway enhances cardiomyocyte survival and angiogenesis, whereas the TGF-β signaling pathway, in synergy with Wnt, drives fibrosis and maladaptive cardiac remodeling [18].

Various therapeutic strategies have been explored to mitigate ischemia-reperfusion injury. Ischemic preconditioning, which involves brief episodes of ischemia before a prolonged ischemic insult, has been shown to reduce infarct size and enhance myocardial resilience. Pharmacological postconditioning, in which specific agents are administered at the onset of reperfusion, has emerged as a more clinically feasible approach. Several pharmacological agents have shown potential in modulating ischemia-reperfusion injury. Glycogen synthase kinase-3 beta (GSK-3β) inhibitors, such as NP12, reduce fibrosis and apoptosis by stabilizing β-catenin in the Wnt signaling pathway. JNK inhibitors, including SP600125, mitigate inflammation and apoptosis by modulating non-canonical Wnt signaling. NF-κB inhibitors, such as dexamethasone, suppress inflammatory cascades, while RAGE inhibitors target endothelial dysfunction and cytokine release [3,7,18].

Epigenetic modifications such as DNA methylation and histone acetylation are implicated in atherosclerosis progression. The TET2 gene has been identified as a regulator of DNA demethylation in vascular endothelial cells, and its overexpression has been associated with reduced plaque formation. Additionally, polymorphisms in genes such as JCAD, SIRT1, and TCF21 influence endothelial integrity and vascular smooth muscle cell (VSMC) proliferation, further modulating CAD risk [3,7,17,19]. VSMCs from the tunica media migrate into the intima and contribute to forming a fibrous cap, stabilizing the plaque. However, activated macrophages also release matrix metalloproteinases (MMPs), which degrade the extracellular matrix, weakening the fibrous cap and increasing the risk of rupture [14,19]. In some cases, atherosclerotic plaques remain stable and gradually obstruct blood flow, leading to chronic stable angina. However, plaques with thin fibrous caps and large necrotic cores are highly vulnerable to rupture. When a plaque ruptures, the exposure of its thrombogenic lipid core to circulating blood results in platelet activation, aggregation, and thrombus formation, leading to acute coronary syndromes (ACS), including unstable angina, non-ST elevation myocardial infarction (NSTEMI), and ST-elevation myocardial infarction (STEMI) [15]. The severity of the ischemic insult depends on the degree and duration of coronary occlusion. Partial obstruction results in subendocardial ischemia, whereas complete occlusion can lead to transmural myocardial infarction and subsequent heart failure [16].

The clinical presentation of CAD varies depending on the extent and stability of atherosclerotic lesions. Some patients remain asymptomatic for decades, while others develop progressive ischemic symptoms. The major clinical manifestations include the following:

- Stable angina: Predictable exertional chest pain caused by fixed coronary stenosis leading to supply–demand mismatch in myocardial oxygenation.
- Unstable angina: Increased plaque instability and thrombosis result in worsening ischemic symptoms, often at rest.
- Myocardial infarction (MI): Complete occlusion of a coronary artery leads to myocardial necrosis.
- Ischemic cardiomyopathy: Chronic ischemia contributes to left ventricular dysfunction and heart failure.

Coronary angiography remains the gold standard for diagnosing CAD, providing anatomical visualization of coronary stenosis. However, functional imaging modalities such as myocardial perfusion imaging (MPI), cardiac magnetic resonance (CMR), and fractional flow reserve (FFR) help assess the physiological significance of coronary lesions [4,16]. Beyond conventional pharmacological therapy (statins, antiplatelets, and beta-blockers) and interventional approaches (PCI and CABG), regenerative medicine offers novel therapeutic strategies for CAD management. MSCs, derived from sources such as bone marrow, adipose tissues, and umbilical cords, possess unique properties, including self-renewal, multipotent differentiation, and immunomodulation, making them ideal for cardiovascular repair. As advancements in regenerative medicine continue, MSCs hold significant potential for integration into standard CAD and IHD treatment paradigms [20].

## 3. Mesenchymal Stem Cells

MSCs are multipotent, non-hematopoietic progenitor cells capable of self-renewal and differentiation into various cell lineages. They were first identified by Friedenstein and colleagues in 1966, and since then, their potential for tissue regeneration, immunomodulation, and paracrine signaling has been widely recognized [21]. MSCs can be derived from multiple sources, including bone marrow, adipose tissue, umbilical cord, and placenta [22,23,24]. While some studies suggest that MSCs from different sources may exhibit distinct biological properties that could influence their regenerative capacity, many of these differences may stem from variability in study design, donor characteristics, isolation and culture methods, and measurement techniques, as well as potential publication bias. Nevertheless, from a practical standpoint, adipose tissue is often considered advantageous due to its higher cell yield and ease of harvest. Their therapeutic efficacy is predominantly attributed to paracrine-mediated signaling, immunomodulatory interactions, pro-angiogenic activity, and extracellular vesicle secretion rather than direct lineage-specific differentiation into target tissues. These properties make MSCs a promising tool for regenerative medicine, including applications in CAD [6,23,25,26].

BM-MSCs have been the most extensively studied. Their invasive extraction procedure and limited proliferation capacity have led researchers to explore alternative sources [27]. AD-MSCs are easily accessible and exhibit strong immunomodulatory properties, making them ideal candidates for cell-based therapies [21]. UC-MSCs and placental MSCs (P-MSCs) are considered more primitive and exhibit enhanced regenerative potential with lower immunogenicity, making them particularly attractive for allogeneic transplantation [22].

**Figure 2:**
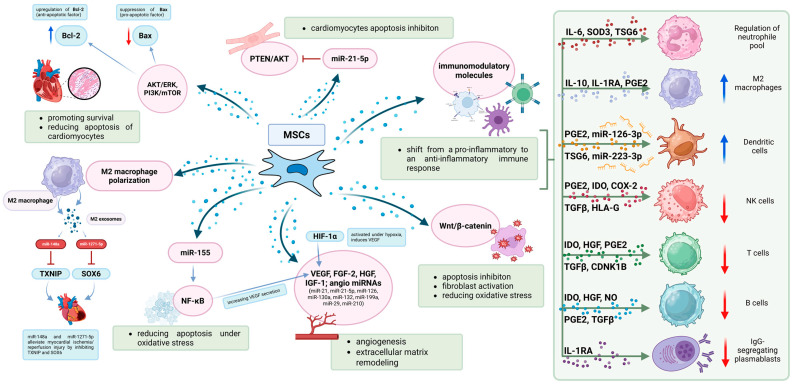
Mechanisms by which MSCs exert their therapeutic effects. MSCs play a key role in modulating immune responses and supporting heart tissue repair. Through the secretion of cytokines, exosomes, and microRNAs, MSCs promote M2 macrophage polarization, inhibit apoptosis, reduce oxidative stress, and stimulate angiogenesis. They help shift the immune response from pro-inflammatory to anti-inflammatory by influencing various immune cells, regulating neutrophils, and enhancing anti-inflammatory macrophages and dendritic cells, while suppressing NK cells, T cells, B cells, and plasmablasts via immunomodulatory molecules. MSCs also activate protective signaling pathways (e.g., AKT/ERK/PI3K/mTOR and Wnt/β-catenin) and enhance cardiomyocyte survival. Blue arrows indicate stimulatory effects, while red arrows indicate inhibitory effects. Red vertical lines with final strikethrough additionally represent a complete inhibition or termination of a given pathway or molecular interaction.

One of the most important mechanisms by which MSCs exert their therapeutic effects is through the secretion of bioactive molecules that regulate the cellular microenvironment and enhance tissue repair (Figure 2). MSCs release various growth factors and cytokines that promote angiogenesis, cardioprotection, and extracellular matrix remodeling [28]. Some of the most critical secreted factors include VEGF, which promotes endothelial cell proliferation and neovascularization, improving myocardial perfusion; FGF-2 and IGF-1, which enhance angiogenesis, cell survival, and extracellular matrix remodeling; and HGF, which exerts anti-fibrotic effects by inhibiting excessive extracellular matrix deposition and preventing scar formation [20]. Under hypoxic conditions, MSCs upregulate HIF-1α, which enhances VEGF expression and further stimulates capillary formation in ischemic tissues [28]. MSCs secrete extracellular vesicles, including exosomes, which mediate cell-to-cell communication by transferring proteins, lipids, and nucleic acids to recipient cells. Among the most critical regulatory molecules contained within MSC-derived exosomes are microRNAs (miRNAs), which modulate gene expression and impact inflammation, apoptosis, and angiogenesis [6,25].

Specific MSC-derived exosomal miRNAs have been identified as key regulators of myocardial repair. For instance, miR-126 and miR-210 enhance endothelial cell proliferation and promote capillary formation; miR-21, particularly miR-21-5p, inhibits cardiomyocyte apoptosis via the PTEN/Akt pathway, thereby increasing cell survival; and miR-199a and miR-29 modulate fibroblast activity and reduce myocardial fibrosis. Notably, several of these miRNAs, such as miR-126, miR-130a, miR-132, and miR-210, are categorized as angiomiRs, a class of microRNAs known to regulate angiogenesis [29]. Explicitly recognizing their pro-angiogenic function helps to unify their collective role in mediating the vascular effects of MSC-derived exosomes. Recent evidence further supports these findings: Bhaskara et al. reported that MSC-derived exosomal miR-21-5p contributes to cardiac repair by regulating PTEN and apoptotic signaling pathways, while miR-126, enriched in MSC exosomes, promotes endothelial proliferation and tube formation [30]. MSC-derived exosomes are being explored as a cell-free therapeutic alternative, potentially offering the regenerative benefits of MSCs while minimizing risks such as immunogenicity and tumorigenicity [31,32,33,34]. However, unlike viable MSCs, exosomes lack the capacity to dynamically sense and respond to microenvironmental cues, which may limit their ability to fully emulate the adaptive therapeutic functions of their parent cells. This distinction highlights both the advantages and the constraints of exosome-based therapies.

As an example of an engineered MSC therapy, Bai et al. investigated the therapeutic potential of MSCs overexpressing MIR155HG in mitigating vascular intimal hyperplasia, a major contributor to graft failure following CABG. MIR155HG functions as a competing endogenous RNA (ceRNA) for miR-205, enhancing MSC-mediated endothelial repair while inhibiting smooth muscle proliferation and fibrosis. In vitro analyses demonstrated that MIR155HG-MSCs exhibited enhanced proliferative and migratory capacity, reduced apoptosis under oxidative stress, and increased VEGF secretion via NF-κB pathway activation. In a rat vein graft model, MIR155HG-MSCs significantly attenuated intimal thickening and collagen deposition [35]. These findings suggest that MIR155HG modulation enhances MSC therapeutic efficacy, providing a promising strategy for improving long-term graft patency post-CABG.

MSCs have potent immunosuppressive properties, which play a key role in modulating post-ischemic inflammation. They interact with immune cells, including macrophages, T cells, and dendritic cells, shifting the immune response from a pro-inflammatory to an anti-inflammatory state [28]. A critical immunomodulatory mechanism of MSCs is their ability to polarize macrophages from the pro-inflammatory M1 phenotype to the reparative M2 phenotype. This shift is mediated by the secretion of IL-10, TGF-β, and PGE2, which enhance tissue repair and prevent excessive post-ischemic inflammation [8,20]. Additionally, MSCs inhibit the activation and proliferation of T cells through the secretion of indoleamine 2,3-dioxygenase (IDO) and galectin-1, both of which suppress pro-inflammatory responses and promote immune tolerance [36].

MSCs exert direct cytoprotective effects on cardiomyocytes, promoting survival and reducing apoptosis through key intracellular signaling pathways. They activate Akt, ERK1/2, and PI3K/mTOR, leading to the upregulation of Bcl-2 (anti-apoptotic protein) and the suppression of Bax [pro-apoptotic factor] [28,37]. Moreover, MSCs mitigate oxidative stress by enhancing the activity of antioxidant enzymes such as SOD and catalase, which neutralize reactive oxygen species (ROS) and protect cardiomyocytes from oxidative damage [25].

One of the challenges in MSC therapy is ensuring effective homing and retention in ischemic tissues. MSC migration is largely regulated by the SDF-1/CXCR4 axis, which directs them toward the injured myocardium [27]. However, a significant proportion of transplanted MSCs fail to engraft due to immune clearance and the harsh ischemic microenvironment. Strategies such as hypoxic preconditioning, genetic modifications, and combination with biomaterial scaffolds are being explored to enhance MSC survival and retention in the heart [28,38]. Additionally, exosome-based therapies, which utilize the bioactive vesicles secreted by MSCs, have emerged as a promising alternative to direct cell transplantation, offering similar regenerative benefits with reduced risk of immune rejection [21,34].

Several clinical trials have investigated MSC-based therapies in CAD, demonstrating improvements in left ventricular ejection fraction (LVEF), myocardial perfusion, and infarct size reduction [38]. Meta-analyses suggest that MSC therapy is most effective in patients with a lower baseline LVEF (8 < 50%), with optimal administration occurring 3–7 days post-MI [39]. With continued advancements, MSC therapy can potentially transform the treatment landscape of CAD, improving patient outcomes and reducing cardiovascular mortality.

## 4. Current Clinical Applications of MSCs in CAD

MSC therapy has emerged as a promising regenerative approach for CAD, particularly in patients who are not candidates for revascularization strategies such as PCI or CABG. Numerous clinical trials have been conducted to evaluate the safety, efficacy, and mechanistic benefits of MSC-based interventions. These studies have assessed critical parameters such as MSC source, dose levels, administration routes, and the use of fresh versus cryopreserved cells while also evaluating primary and secondary endpoints to determine clinical efficacy (Table 1).

### 4.1. Bone-Marrow-Derived Mesenchymal Stem Cell Therapy in CAD

BM-MSCs have emerged as one of the most extensively studied cell sources for cardiac regeneration due to their immunomodulatory properties, angiogenic potential, and ability to enhance myocardial function through paracrine signaling. Multiple clinical trials have investigated their therapeutic effects, particularly in patients with ischemic heart failure (IHF) CAD, where standard revascularization options are unavailable [40,41,66,67]. The studies collectively demonstrate that BM-MSCs hold significant therapeutic potential in treating various forms of heart failure. Their mechanism of action is predominantly paracrine, involving the secretion of bioactive molecules that stimulate endogenous repair, reduce fibrosis, and modulate immune responses. A systematic review and meta-analysis of phase II/III randomized clinical trials (RCTs) evaluating BM-MSCs in heart failure patients concluded that MSC therapy improves LVEF by approximately 6.37% and increases the six-minute walking distance (6MWD) by 27.86 m compared to controls. These results indicate a functional benefit but also highlight the need for larger, more robust trials to establish clinical guidelines for their routine use [67]. A randomized, placebo-controlled study on ischemic cardiomyopathy found that transendocardial injections of MSCs reduced infarct size by 18.9% and improved regional myocardial function, although they did not significantly affect left ventricular volume or global ejection fraction. Importantly, MSCs showed superior anti-fibrotic and pro-regenerative effects compared to bone marrow mononuclear cells [40].

In nonischemic dilated cardiomyopathy, a trial comparing autologous versus allogeneic MSCs found that allogeneic MSCs led to an 8.0% improvement in LVEF and a 37-m increase in the 6MWD, whereas autologous MSCs provided only a 5.4% LVEF improvement and no significant functional gains. This suggests allogeneic MSCs may be a more viable off-the-shelf therapeutic option due to their immunomodulatory properties [42]. Another study using autologous BM-MSC injections in severe ischemic heart failure demonstrated a significant reduction in Left Ventricular End-Systolic Volume (LVESV) and an increase in stroke volume and myocardial mass, confirming the regenerative potential of MSCs. However, no major differences in functional capacity were observed, indicating that structural improvements may not always translate directly to symptomatic relief [41]. A broader review of MSC-based cardiovascular therapy emphasized that MSCs not only reduce myocardial scar size and improve tissue perfusion but also regulate immune responses and inhibit fibrosis. Strategies to enhance MSC efficacy, such as genetic modifications or combination therapies with biomaterials, are being explored to optimize their therapeutic impact [66]. The DREAM-HF Phase III trial, one of the largest and most comprehensive studies on cell therapy for heart failure, evaluated allogeneic bone marrow-derived mesenchymal precursor cells (MPCs) in patients with heart failure with reduced ejection fraction (HFrEF). While the primary endpoint was not met, MPC therapy significantly reduced the risk of myocardial infarction or stroke by 58% and by 75% in patients with elevated inflammation (hsCRP ≥ 2 mg/L). A modest improvement in LVEF and a reduction in major adverse cardiovascular events were also observed, suggesting potential benefits, particularly in inflammation-driven heart failure [43]. Collectively, these studies reinforce the safety and regenerative potential of BM-MSC-based therapy in cardiovascular disease.

### 4.2. Adipose-Derived Mesenchymal Stem Cell Therapy in CAD

AD-MSCs have emerged as a promising alternative to BM-MSCs due to their abundance, ease of isolation, and potent regenerative properties. Their therapeutic effects in ischemic heart disease are primarily mediated through paracrine signaling, modulating immune responses, enhancing angiogenesis, and attenuating fibrosis. Several preclinical and clinical studies have investigated their efficacy in treating MI and heart failure, particularly in patients without revascularization options [44,68,69].

A systematic review of stem cell therapy in AMI patients showed sustained LVEF improvement up to 36 months and a trend toward reduced major adverse cardiac events risk, especially with longer cell culture durations and higher cell doses. Specifically, studies with cell culture durations longer than one week demonstrated significant LVEF increases of 4.32% at 6 months, 1.89% at 12 months, and 5.23% at 24 months. In contrast, studies with cell culture periods of one week or less showed significant improvement only at 6 months. However, infarct size reduction was not significant [70]. Another systematic review similarly highlighted the limited but promising evidence regarding the efficacy of AD-MSCs in patients with ischemic heart disease [71]. Myocardial scintigraphy studies demonstrated a significant reduction in stress-induced ischemia exclusively in AD-MSC-treated patients [45,46]. Although overall left ventricular function did not significantly differ between groups, one study reported improved parietal motility in treated segments using cardiac MRI [46]. Functional capacity increased in AD-MSC-treated patients in at least three studies [45,46,47], and one study [45] documented subjective improvements in angina functional class and heart failure symptoms. Additionally, a reduction in the post-infarction fibrosis area, indicating extracellular matrix remodeling, was observed in two studies [46,48]. Most of the included trials were early phase [phase I or II] with small sample sizes and primarily reported surrogate endpoints, limiting their statistical power to detect significant differences. Although trends favored the AD-MSC group in some outcomes, the two most recent trials, DANISH [44] and SCIENCE [49], confirmed safety but showed disappointing results in terms of efficacy, potentially due to the use of standardized allogeneic cell products.

The angiogenic potential of AD-MSCs has also been well-documented. Animal studies have shown that AD-MSCs secrete high levels of VEGF, HGF, and stromal cell-derived factor 1 (SDF-1) promoting neovascularization and improving myocardial perfusion [50,51,52].

### 4.3. Umbilical-Cord-Derived Mesenchymal Stem Cell Therapy in CAD

UC-MSCs have gained attention due to their low immunogenicity. UC-MSCs possess strong anti-inflammatory and pro-angiogenic properties, making them a promising candidate for ischemic tissue repair. Compared to BM-MSCs and AD-MSCs, UC-MSCs exhibit superior proliferative capacity [39]. One study assessed the safety and feasibility of injecting UC-MSCs into the epicardial coronary arteries that supply collateral circulation in elderly patients with chronic total coronary occlusion. Participants were randomly assigned to one of three dosing groups: low-dose (3 × 10^6^ cells), mid-dose (4 × 10^6^ cells), and high-dose (5 × 10^6^ cells). Myocardial perfusion was assessed using 99mTc single-photon emission computed tomography (SPECT) at 12 and 24 months. The results showed a significant reduction in infarct size and a notable improvement in LVEF [53].

### 4.4. Animal Models

MSC-based therapies have demonstrated significant cardioprotective effects across various preclinical and experimental models of myocardial ischemia, chronic total occlusion, and inflammatory coronary injury. In a murine model of Kawasaki disease, human UC-MSCs (hUC-MSCs) effectively reduced coronary artery lesions, inflammatory infiltration, and fibrosis, suggesting their potential as an anti-inflammatory and cardioprotective therapy for coronary artery injury [54]. Similarly, MSC-based interventions in MI models have shown improvements in cardiac function, as evidenced by increased LVEF, reduced fibrosis, and enhanced myocardial regeneration. Direct injection of AD-MSCs into infarcted myocardium improved myocardial viability and upregulated cardiac transcription factors, while fibrin-encapsulated exosome-based therapy (hEnMSCs-EXOs) promoted angiogenesis and attenuated fibrosis, further confirming the regenerative potential of MSC-derived exosomes [55,56]. Systematic analyses of amniotic membrane-derived MSCs (AM-MSCs) in ischemic cardiomyopathy models also reinforce these findings, with meta-analysis revealing significant improvements in LVEF and reductions in myocardial fibrosis, despite heterogeneity in study designs [72]. Given the inherent limitations of native MSCs, genetically modified MSCs have been explored as an advanced strategy to enhance survival, differentiation potential, and angiogenic capacity. Genetic modifications, including overexpression of pro-survival genes (Akt1 and Bcl-xL) and angiogenic factors (VEGF and Angiopoietin-1), have been shown to improve myocardial engraftment, vascularization, and overall cardiac recovery post-MI, with functional benefits reflected in higher LVEF and reduced infarct size [73]. Additionally, MSC-based adjuncts to surgical interventions, such as MSC-loaded patches used alongside CABG, have demonstrated improved diastolic function, reduced fibrosis, and decreased inflammatory cytokine expression in a porcine model of hibernating myocardium. The observed increase in PGC1α expression suggests enhanced mitochondrial function and energy homeostasis, further supporting the regenerative capacity of MSC therapy in ischemic heart disease [57]. Collectively, these studies highlight MSC-based therapies as a multifaceted approach to cardiac repair, offering immunomodulation, fibrosis attenuation, angiogenesis promotion, and myocardial regeneration. While preclinical data strongly support their efficacy, further research is needed to optimize administration strategies, refine genetic modifications, and translate these findings into standardized clinical protocols for human application. However, animal models may not fully predict clinical efficacy due to species differences, challenges in modeling human comorbidities and immune responses, and the use of higher MSC doses or delivery routes that may not be feasible in humans [54,55,56,57,72,73].

## 5. CD34^+^ Stem Cells

CD34^+^ stem cell therapy has been predominantly investigated in patients with refractory angina and diffuse triple-vessel coronary artery disease who are not eligible for PCI or CABG. A representative study conducted at the General Hospital of Beijing Military Region enrolled 112 patients with Canadian Cardiovascular Society (CCS) class III–IV angina, all of whom remained symptomatic despite optimal medical therapy. Autologous CD34^+^ cells, enriched from bone marrow aspirates using the CliniMACS selection system, were administered via intracoronary infusion. The treatment group demonstrated a marked and sustained reduction in angina frequency, from 21.2 to 5.6 episodes per week at six months, compared to minimal improvement in the control group. Moreover, a significant proportion of treated patients experienced at least a one-class improvement in CCS angina grading, paralleled by perfusion improvements on SPECT imaging and no increase in arrhythmic events on 24-h Holter monitoring [10]. While mechanistically distinct from MSCs, CD34^+^ cells, primarily comprising endothelial progenitor cells, share overlapping therapeutic targets such as neovascularization, endothelial repair, and microvascular perfusion enhancement. These findings underscore the broader clinical relevance of cell-based therapies in ischemic heart disease and provide context for ongoing investigations into MSC-mediated approach [58,74,75].

### Comparison to MSC-Based Approaches

While BM-MSCs, AD-MSCs, and UC-MSCs exert their therapeutic effects primarily through paracrine signaling, immunomodulation, and cardioprotection, CD34^+^ cells function via a direct endothelial reparative mechanism. Unlike traditional MSCs, which support myocardial regeneration by mitigating inflammation and apoptosis, CD34^+^ cells actively promote neovascularization and enhance microvascular perfusion. This mechanistic distinction positions CD34^+^ cell therapy as a particularly promising strategy for patients with advanced ischemic coronary artery disease and prominent microvascular dysfunction [10]. Given their complementary modes of action, MSCs and CD34^+^ cells may not be mutually exclusive but rather components of a broader regenerative paradigm. A future-oriented approach could involve tailoring cell therapy based on dominant pathophysiological features, prioritizing MSCs in inflammation-driven cardiomyopathies and CD34^+^ cells in cases characterized by microvascular rarefaction. Although clinical studies combining both cell types are currently lacking, this conceptual framework opens the door to individualized or sequential therapeutic strategies designed to optimize patient-specific outcomes.

## 6. Administration Strategies of MSC Therapy

The administration of MSCs for therapeutic purposes in cardiovascular disease, particularly in IHD and CAD, has been extensively explored through various delivery methods. Each approach offers unique advantages and limitations, influencing MSC retention, engraftment, therapeutic efficacy, and overall clinical outcomes. The primary methods of MSC administration include intravenous infusion, intracoronary injection, intramyocardial injection, subcutaneous transplantation, and cell sheet transplantation. The choice of method is dictated by factors such as the targeted myocardial region, the extent of ischemic injury, the immunological compatibility of MSCs, and the feasibility of delivery in clinical settings.

### 6.1. Intravenous Infusion of MSCs

Intravenous infusion (IV) via a central venous catheter remains one of the most investigated routes for MSC therapy due to its non-invasive nature and ease of administration [10,59]. Upon systemic infusion, MSCs distribute themselves through the vascular system and home to injury sites via mechanisms involving chemokine signaling (e.g., via the stromal-derived factor-1 or SDF-1/CXCR4 axis). However, subjected to the first-pass effect, a significant proportion of MSCs become trapped in the lungs, reducing the number of cells reaching the ischemic myocardium [60]. While IV administration has demonstrated some efficacy in reducing inflammation and promoting angiogenesis, its therapeutic benefits for cardiac regeneration remain inferior compared to direct myocardial delivery [60]. This aligns with preclinical findings in a mouse model of colitis, where IV MSCs failed to improve outcomes, unlike subcutaneous or intraperitoneal routes, which demonstrated superior efficacy and in vivo persistence [59].

### 6.2. Intracoronary Injection of MSCs

Intracoronary (IC) infusion of MSCs has emerged as a promising therapeutic strategy for acute myocardial infarction (AMI), facilitating targeted delivery to ischemic myocardial regions to promote cardiac repair. While early studies, such as the TOPCARE-AMI trial, confirmed the long-term safety of IC BM-MSC infusion with modest improvements in LVEF, challenges such as low cell retention and potential microvascular obstruction remain limitations [10]. However, more recent studies—employing alternative MSC sources, such as Wharton’s jelly-derived MSCs which improved delivery techniques, and even dual-route administration, involving both intracoronary and intravenous injection —have demonstrated encouraging advances in both safety and therapeutic efficacy [61,62].

Several studies have confirmed the efficacy of IC MSC administration in improving cardiac function and ventricular remodeling. A randomized, double-blind, multicenter study demonstrated that IC infusion of Wharton’s jelly-derived MSCs (WJ-MSCs), a type of MSC isolated from the connective tissue of the umbilical cord, significantly increased LVEF and reduced left ventricular end-systolic and end-diastolic volumes, suggesting favorable reverse remodeling. Importantly, no increased risk of arrhythmias, immune reactions, or microvascular obstruction was observed, highlighting the safety of this approach [61]. Similar findings were reported in patients receiving IC transplantation of BM-MSCs via a non-infarct-related artery, where improvements in myocardial perfusion, cardiomyocyte viability, and left ventricular function were confirmed through imaging techniques such as 18F-deoxyglucose SPECT and 99mTc-MIBI [63]. A novel dual-route MSC administration strategy, combining IC and IV infusion, has further improved therapeutic efficacy by leveraging both localized myocardial regeneration and systemic immunomodulation. In one study, IC injection was followed by IV MSC infusion, extending the therapeutic window and enhancing myocardial recovery. Patients receiving this combined therapy exhibited significant improvements in LVEF, reduced NT-proBNP levels, and enhanced regional wall motion, with no major adverse events such as coronary occlusion, immune reactions, or pulmonary complications [62]. Collectively, these findings reinforce the potential of IC MSC therapy as a regenerative approach for AMI, with multiple studies demonstrating improvements in myocardial function, ventricular remodeling, and perfusion. The combination of IC and IV administration appears to enhance therapeutic outcomes by prolonging the regenerative effects of MSCs while maintaining safety. However, further research is needed to optimize dosing strategies, improve cell retention, and validate long-term clinical benefits through larger, multicenter trials [10,61,62,63].

### 6.3. Intramyocardial Injection of MSCs

Intramyocardial (IM) injection is a direct and targeted approach for MSC delivery, offering high cell retention by preventing immediate washout into circulation while ensuring precise localization within ischemic or peri-ischemic regions. This method is particularly beneficial for patients with chronic ischemic heart disease or non-revascularized myocardium, where vascular-based delivery techniques may be less effective [10,76]. Studies consistently show that IM MSC administration enhances angiogenesis, reduces fibrosis, and improves cardiac function, particularly in patients with severe left ventricular dysfunction. However, despite these advantages, the invasive nature of the procedure carries potential risks, including arrhythmia, myocardial injury, and procedural complications, and its long-term efficacy is influenced by the hostile ischemic microenvironment, which limits cell survival [10,64,76].

Although Rodrigo et al. reported no significant arrhythmias, other studies have noted this as a possible complication, particularly in patients with advanced ventricular dysfunction [10,64,76]. Additional risks include microvascular disruption, aberrant vascularization, or intramyocardial hemorrhage—especially if injection sites are not optimally selected. Moreover, poor cell survival within the ischemic microenvironment remains a major limitation, with oxidative stress, hypoxia, and inflammation contributing to apoptotic cell loss and reduced long-term efficacy [64,76]. Immune reactions are another consideration, particularly in the context of allogeneic MSCs, which, despite their immunomodulatory properties, may still trigger localized rejection. Autologous MSCs avoid this issue but require ex vivo expansion, potentially introducing variability in cell quality and therapeutic outcomes [5]. While both autologous and allogeneic MSCs require ex vivo expansion, the implications differ; autologous MSCs are associated with variability in cell potency and therapeutic outcomes due to donor-specific factors, whereas allogeneic MSCs, despite enabling standardization, may face challenges related to overexpansion and phenotypic drift during large-scale manufacturing [5].

### 6.4. Subcutaneous Transplantation of MSCs

An emerging strategy for MSC delivery involves subcutaneous (SC) transplantation, which circumvents the pulmonary entrapment seen in IV infusion while avoiding the invasive nature of IC or IM approaches. This method allows MSCs to form multicellular aggregates, enhancing their paracrine secretion of trophic factors over an extended period. Experimental studies suggest subcutaneously transplanted MSCs can modulate systemic inflammatory responses, promote remote angiogenesis, and provide cardioprotective effects via cytokine signaling [60]. This is further supported by preclinical evidence in a murine model of colitis, where subcutaneous delivery of MSCs led to significant improvements in disease activity and histological outcomes. Compared to IV administration, SC-transplanted MSCs demonstrated prolonged in vivo persistence and greater therapeutic efficacy, reinforcing their potential for systemic immunomodulation and regenerative effects [59]. However, SC administration is less commonly utilized in clinical settings, as its direct impact on myocardial repair remains less well understood compared to IC and IM methods.

### 6.5. Cell Sheet Transplantation of MSCs

Cell sheet transplantation represents a scaffold-free tissue engineering approach, where MSCs are cultured as monolayers and transferred as intact sheets onto the myocardial surface. This method enhances MSC survival, engraftment, and retention, avoiding the issue of poor cell persistence observed with IC and IM injections. Preclinical studies have demonstrated that MSC sheets significantly improve cardiac function by promoting vascularization, suppressing fibrosis, and enhancing electrical coupling with host cardiomyocytes [76]. Moreover, UC-MSC sheets, due to their low immunogenicity and ease of isolation, have been proposed as a promising candidate for allogeneic transplantation in ischemic heart disease. Despite these advantages, challenges remain regarding standardization of sheet fabrication, long-term viability, and optimal transplantation techniques [76].

### 6.6. Bioengineered Scaffolds for MSC Delivery

Recent advancements in tissue engineering have introduced bioengineered scaffolds as promising platforms for the targeted and sustained delivery of mesenchymal stem cells (MSCs) to ischemic myocardium. These scaffolds, composed of natural or synthetic biomaterials such as collagen, fibrin, or biodegradable polymers, provide structural support and a conducive microenvironment that enhances MSC survival, retention, and paracrine activity post-transplantation. By mimicking aspects of the extracellular matrix, scaffolds facilitate cell–matrix interactions, promote angiogenesis, and modulate local immune responses. Importantly, bioactive scaffolds can serve not only as delivery vehicles but also as modulators of stem cell activation and proliferation, thereby enhancing therapeutic efficacy in cardiac tissue repair [77].

## 7. Fresh vs. Cryopreserved MSCs

The choice between freshly expanded and cryopreserved MSCs has significant implications for cell viability, potency, and therapeutic efficacy, particularly in regenerative medicine and cardiovascular applications. A systematic review analyzing 18 preclinical studies found no statistically significant differences between fresh and cryopreserved MSCs in most in vivo efficacy outcomes, with only 2.3% of animal experiments showing significant differences, and these findings were inconsistent, with some favoring fresh MSCs, while others favored cryopreserved cells. Similarly, in vitro assays reported functional differences in only 13% of cases, indicating that cryopreservation does not substantially impair MSCs’ regenerative or immunomodulatory properties [78]. As the Dave et al. review notes, the use of xenogeneic MSCs in immunocompetent animals, non-physiological dosing, and other model-specific limitations may obscure subtle but clinically relevant differences in therapeutic potency [78]. Further investigation into how syngeneic and xenogeneic MSCs differentially influence inflammatory disease models, particularly in relation to HLA expression, co-stimulatory signaling, paracrine activity, and species-specific cytokine–receptor interactions, may provide critical insights to enhance the translational potential of MSC-based therapies in human clinical trials [78]. A study comparing freshly cultured MSCs with those derived from frozen bone marrow mononuclear cells (BM-MNCs) or expanded and cryopreserved MSCs found no significant differences in proliferation capacity, differentiation potential, or endothelial function. Both fresh and cryopreserved MSCs exhibited comparable ability to differentiate into endothelial-like cells and form vascular structures in vitro, with similar VEGF-induced gene expression and tube formation capabilities. Flow cytometry confirmed that MSCs retained their immunophenotypic characteristics after cryopreservation, though minor reductions in CD73, CD90, and CD166 expression were noted, suggesting minimal phenotypic alterations [65].

These findings reinforce the viability of MSC banking, allowing for standardized and scalable cell therapy applications for ischemic heart disease and other degenerative conditions [65,77,78]. Despite these findings, some studies suggest that freshly cultured MSCs may have advantages in terms of proliferation rate, paracrine signaling, and differentiation capacity. Sid-Otmane et al. [79] demonstrated that fresh MSCs exhibit stronger regenerative potential, while cryopreservation can induce cellular stress, reduce membrane integrity, and impair immunomodulatory functions. However, preconditioning strategies, such as exposure to growth factors or hypoxic environments before administration, have been proposed to mitigate these effects and enhance post-transplantation survival [80]. Overall, the evidence suggests that cryopreserved MSCs maintain substantial therapeutic efficacy, making them viable for off-the-shelf clinical applications. However, where immediate regenerative capacity is critical, such as in acute cardiovascular events, freshly expanded MSCs may offer superior outcomes. Further research is needed to optimize cryopreservation techniques and preconditioning strategies to enhance the functional properties of stored MSCs [65,78,79,80].

## 8. Conclusions

MSC therapy has emerged as a promising regenerative strategy in the treatment of CAD, particularly in patients with diffuse, non-revascularizable ischemic cardiomyopathy. Unlike conventional revascularization approaches such as PCI or CABG, which primarily address epicardial flow restoration, MSCs target the molecular and cellular hallmarks of CAD pathology—including endothelial dysfunction, chronic inflammation, microvascular rarefaction, and maladaptive remodeling. Their therapeutic effects are predominantly mediated via paracrine mechanisms, encompassing the secretion of angiogenic factors (e.g., VEGF, FGF-2, and HGF), anti-apoptotic and anti-fibrotic cytokines, as well as extracellular vesicles rich in regulatory microRNAs (e.g., miR-21-5p, miR-126, and miR-210). These molecules collectively orchestrate myocardial repair by promoting neovascularization, enhancing cardiomyocyte survival, modulating immune cell polarization, and attenuating fibrosis.

Both preclinical and clinical studies have demonstrated modest but reproducible improvements in surrogate endpoints such as LVEF, infarct size reduction, myocardial perfusion, and symptomatic status—especially in patients with severely reduced baseline LVEF and heightened systemic inflammation. Comparative data across MSC sources suggest biological variability in immunomodulatory and angiogenic potency, with UC-MSCs offering advantages in proliferative capacity and allogeneic applicability, while AD-MSCs present logistical benefits due to high yield and ease of harvest. Moreover, preclinical studies have supported the additive role of exosome-based therapies and genetically engineered MSCs, which enhance survival and paracrine potency under ischemic stress.

The efficacy of MSC therapy is also influenced by the route of administration. IM injection, although invasive, provides high local cell retention and has shown superior effects on regional function. IC infusion offers targeted delivery to peri-infarct zones, but is limited by risks of microvascular obstruction and low engraftment. IV delivery benefits from immunomodulatory reach but suffers from pulmonary sequestration. Novel delivery modalities—such as MSC-loaded scaffolds, engineered exosome formulations, and dual-route (IC+IV) strategies—are under investigation to optimize biodistribution and therapeutic impact. Notably, MSC-derived exosomes may serve as a cell-free alternative, preserving many regenerative functions while minimizing immunogenic and tumorigenic risks. However, their lack of dynamic environmental responsiveness remains a limiting factor compared to viable MSCs.

Despite significant progress, several translational challenges persist. These include low long-term engraftment and survival of transplanted cells, variability in manufacturing protocols, and inter-patient heterogeneity in response. The debate between fresh and cryopreserved MSCs remains unresolved, as subtle differences in viability, secretome composition, and immunomodulatory activity may interact with route-specific pharmacodynamics. Furthermore, the limitations of current preclinical models—particularly species differences in immune responses, MSC kinetics, and disease comorbidities—warrant caution in extrapolating animal data to human trials.

Looking ahead, the field is moving toward precision-based regenerative therapy. Future strategies may involve stratifying patients based on key biological parameters such as inflammatory status, ischemic burden, and comorbidities, as well as selecting specific cell sources or delivery routes tailored to the dominant pathophysiological mechanism. For example, MSCs may be prioritized in inflammation-driven cardiomyopathy, while CD34^+^ endothelial progenitor cells may be more effective in microvascular dysfunction. The potential for sequential or combinatorial cell therapy paradigms—guided by biomarker-driven patient profiling—represents a rational framework for maximizing therapeutic efficacy while minimizing risks.

In conclusion, MSC therapy may not yet represent a paradigm shift in CAD management, but it is increasingly positioned to complement and extend existing strategies by targeting biological processes underlying myocardial injury and repair. With continued advancements in cell manufacturing, delivery technologies, and clinical trial design, MSC-based therapies are poised to play an integral role in the evolution of personalized cardiovascular regenerative medicine.

## Figures and Tables

**Table 1:** Summary of preclinical, clinical, and experimental studies investigating the therapeutic potential of mesenchymal stem cells (MSCs) and related stem cell types in cardiovascular disease. The table includes study design, stem cell source, delivery methods, reported outcomes, and limitations for each study.

No.	Author(s)	Year	Study Title	Study Type	MSC Source	Delivery Method	Results	Limitations
[11]	Wang et al.	2010	Intracoronary Autologous CD34^+^ Stem Cell Therapy for Intractable Angina	Clinical	Bone Marrow	Intracoronary Injection	Reduction in the frequency of angina episodes per week at 3 and 6 months post-infusion; improvement in nitroglycerine usage, exercise time, CCS class, and myocardial perfusion	Small sample size; single-center recruitment; possible placebo effect of intracoronary infusion alone
[29]	Gong et al.	2017	Mesenchymal Stem Cells Release Exosomes that Transfer Mirnas to Endothelial Cells and Promote Angiogenesis	Preclinical	MSCs line C3H10T1/2 cells purchased from ATCC (Manassas, VA, USA)	-	Conditioned medium increased tube formation and angiogenesis; exosomes mediated miR transfer to HUVECs; pro-angiogenic effects depend on miR cargo	Complex composition of exosomes; comparisons with simple medium/BSA may be inadequate
[32]	Wen et al.	2020	Mesenchymal Stem Cell-derived Exosomes Ameliorate Cardiomyocyte Apoptosis in Hypoxic Conditions Through MicroRNA144 by Targeting the PTEN/AKT Pathway	Preclinical	Bone Marrow	-	MSC-derived exosomes reduce apoptosis in hypoxia via miR-144/PTEN/AKT pathway; cardioprotective effect independent of differentiation	Tested at one time point only; limited generalizability to other hypoxic conditions
[35]	Bai et al.	2024	Improved Therapeutic Effects on Vascular Intimal Hyperplasia by Mesenchymal Stem Cells expressing MIR155HG that Function as a ceRNA for MicroRNA-205	Preclinical	Cyagen Biosciences Inc. (Shanghai, China)	-	MIR155HG improved MSCs viability and migration; acted as sponge for miR-205; enhanced anti-apoptotic and pro-angiogenic function	Further research on MIR155HG needed before clinical application
[40]	Heldman et al.	2014	Transendocardial Mesenchymal Stem Cells and Mononuclear Bone Marrow Cells for Ischemic Cardiomyopathy	Clinical	Bone Marrow	Transendocardial Injection	MSCs were associated with decreasing scar fraction and increasing viable myocardial mass, suggesting true myocardial regeneration; MSCs improved the Minnesota Living With Heart Failure score	Small sample size; not powered to draw efficacy comparisons; multiple comparisons limit conclusions
[41]	Mathiasen et al.	2015	Bone Marrow-derived Mesenchymal Stromal Cell Treatment in Patients with Severe Ischaemic Heart Failure: A Randomized Placebo-controlled Trial (MSC-HF trial)	Clinical	Bone Marrow	Intramyocardial Injection	Significant improvements in LV function (LVESV, LVEF, SV); LV mass and wall thickness improved in treated patients	Adverse events during procedure; underpowered SAE analysis; limited MRI eligibility
[42]	Hare et al.	2017	Randomized Comparison of Allogeneic Versus Autologous Mesenchymal Stem Cells for Nonischemic Dilated Cardiomyopathy	Clinical	Bone Marrow	Transendocardial Injection	Improvements in EF, 6MWT, MLHFQ; allo-MSCs improved endothelial function, TNF-a suppression, NYHA class, MACE, hospitalization rates	No placebo group; patient loss; small sample size limits efficacy interpretation
[43]	Perin et al.	2023	Randomized Trial of Targeted Transendocardial Mesenchymal Precursor Cell Therapy in Patients with Heart Failure	Clinical	Bone Marrow	Transendocardial Injection	No change in nonfatal hospitalization; significant reduction in TTFE for MI or stroke after 30 months	Endpoints may not capture full benefit/mechanism of MPCs
[44]	Qayyum et al.	2023	Danish Phase II Trial using Adipose Tissue Derived Mesenchymal Stromal Cells for Patients with Ischaemic Heart Failure	Clinical	Adipose Tissue	Intramyocardial Injection	No significant change in LV volumes or LVEF; improved quality-of-life and symptoms in ASC group	Safe but no myocardial or clinical improvement
[45]	Qayyum et al.	2019	Autologous Adipose-Derived Stromal Cell Treatment for Patients with Refractory Angina (Mystromalcell Trial): 3-Year Follow-Up Results	Clinical	Adipose Tissue	Intramyocardial Injection	Improved cardiac symptoms in ASC group; exercise capacity unchanged; deterioration observed in placebo group	No significant difference between ASC and placebo groups
[46]	Perin et al.	2014	Adipose-Derived Regenerative Cells in Patients with Ischemic Cardiomyopathy: The PRECISE Trial	Clinical	Adipose Tissue	Transendocardial Injection	Metabolic equivalents and MVO2 preserved in ADRC group; improved LV mass and wall motion; reduced ischemia up to 18 months	Did not reduce scar size or increase LVEF; small sample; baseline MRI/SPECT variability
[47]	Kastrup et al.	2017	Cryopreserved Off-the-Shelf Allogeneic Adipose-Derived Stromal Cells for Therapy in Patients with Ischemic Heart Disease and Heart Failure—A Safety Study	Clinical	Adipose Tissue	Intramyocardial Injection	Improved LV pump function and 6MWT; no procedure-related complications	Used DMSO; no control group; underpowered study
[48]	Houtgraaf et al.	2012	First Experience in Humans Using Adipose Tissue–Derived Regenerative Cells in the Treatment of Patients with ST-Segment Elevation Myocardial Infarction	Clinical	Adipose Tissue	Intracoronary Injection	Safe ADRC infusion; improved cardiac function; reduced scar formation	Small sample; bleeding events during liposuction in 2 patients
[49]	Qayyum et al.	2023	Effect of Allogeneic Adipose Tissue-Derived Mesenchymal Stromal Cell Treatment in Chronic Ischaemic Heart Failure—the SCIENCE Trial	Clinical	Adipose Tissue	Intramyocardial Injection	Safe over 3 years; no significant changes in LVESV, LVEF, or functional markers	Possibly insufficient dose or retention; small adverse events noted
[50]	Zhao et al.	2020	Hypoxic Preconditioning Enhances Cellular Viability and Pro-angiogenic Paracrine Activity: The Roles of VEGF-A and SDF-1a in Rat Adipose Stem Cells	Preclinical	Adipose Tissue	-	Improved protection under hypoxia; upregulation of VEGF-A and SDF-1a pathways	Variable differentiation/survival; optimal hypoxia exposure remains unclear
[51]	Mytsyk et al.	2021	Long-Term Severe In Vitro Hypoxia Exposure Enhances the Vascularization Potential of Human Adipose Tissue-Derived Stromal Vascular Fraction	Preclinical	Adipose Tissue	-	Increased VEGF release and vessel density after hypoxic exposure	High variability; low dividing/apoptotic cell counts; implantation challenges
[52]	He et al.	2015	Hypoxic Adipose Mesenchymal Stem Cells Derived Conditioned Medium Protects Myocardial Infarct in Rat	Experimental	Adipose Tissue	-	HypoCM increased VEGF, HGF, SDF-1; improved cardiomyocyte survival and infarct healing	ADMSCs identity debated; oxygen tension regulation is crucial but unclear
[53]	Li et al.	2015	Safety and Efficacy of Intracoronary Human Umbilical Cord-Derived Mesenchymal Stem Cell Treatment for Very Old Patients with Coronary Chronic Total Occlusion	Clinical	Umbilical Cord	Intracoronary Injection	No major cardiac events in 24 months; reduced infarct size; increased LVEF	Small sample (15 patients)
[54]	Guo et al.	2022	Human Umbilical Cord Mesenchymal Stem Cells Inhibit Coronary Artery Injury in Mice with Lactobacillus casei Wall Extract-Induced Kawasaki Disease	Experimental	Umbilical Cord	-	Reduced coronary artery damage in Kawasaki disease model; improved pathology	Short experiment; poor KD model simulation; dose-dependency unstudied
[55]	Koutela et al.	2024	MSC Transplantation has a Regenerative Effect in Ischemic Myocardium: SPECT-CT Assesment	Experimental	Adipose Tissue	-	Regeneration of ischemic myocardium confirmed by SPECT/CT, histology, and immunohistochemistry	Limited to female donors and male recipients; short monitoring period
[56]	Sepehri et al.	2025	Therapeutic Potential of Exosomes Derived from Human Endometrial Mscs for Heart Tissue Regeneration after myocardial infarction	Experimental	Endometrium	-	Exosomes reduced fibrosis and inflammation; improved cardiac function post-infarction	Low survival and retention of exosomes; mild and short-term effect
[57]	Aggarwal et al.	2023	An Adjuvant Stem Cell Patch with CABG Surgery Improves Diastolic Recovery in Porcine Hibernating Myocardium	Experimental	Bone Marrow	-	MSC patch improved diastolic function, increased PGC1α, reduced inflammation and fibrosis	Juvenile animal model; not representative of advanced atherosclerosis; small CABG+MSC group
[58]	Henry et al.	2022	Autologous CD34^+^ Stem Cell Therapy Increases Coronary Flow Reserve and Reduces Angina in Coronary Microvascular Dysfunction	Clinical	-	Intracoronary Injection	Improved coronary flow reserve, reduced angina, improved CCS class and quality of life; no serious adverse events	No control group; small sample; variation in CD34^+^ delivery; no dose-response observed
[59]	Giri et al.	2020	Mesenchymal stromal cell therapeutic potency is dependent upon viability, route of delivery, and immune match	Experimental	Bone Marrow	-	Subcutaneous/intraperitoneal MSCs effective; heat-inactivated or thawed MSCs lost efficacy; immune match allowed redosing	Cryoinjury may reduce MSC function post-thaw; human translation affected
[60]	Preda et al.	2020	Evidence of mesenchymal stromal cell adaptation to local microenvironment following subcutaneous transplantation	Experimental	Bone Marrow	Subcutaneous Transplantation	MSC aggregates stimulated angiogenesis and protective factors via hypoxia signaling; inflammation noted with high-dose	Cytokine elevation likely reflects host immune response, not MSC effect
[61]	Gao et al.	2015	Intracoronary infusion of Wharton’s jelly-derived mesenchymal stem cells in acute myocardial infarction: Double-blind, randomized controlled trial	Clinical	Wharton’s Jelly (Umbilical Cord)	Intracoronary Injection	Reduced infarct size; improved function and perfusion; prevented adverse LV remodeling	Mechanisms not explored; CE-MRI not universally available; PET used
[62]	Hsiao et al.	2022	First-in-human pilot trial of combined intracoronary and intravenous mesenchymal stem cell therapy in acute myocardial infarction	Clinical	Umbilical Cord	Intracoronary and Intravenous Injections	Improved LVEF and wall motion; NT-proBNP decreased; no major adverse events	Small sample; no placebo group; no immunological marker analysis
[63]	Yang et al.	2010	A Novel Approach to Transplanting Bone Marrow Stem Cells to Repair Human Myocardial Infarction: Delivery via a Noninfarct-relative Artery	Clinical	Bone Marrow	Intracoronary Injection	Improved cardiac function and perfusion 6 months post-treatment; safe and feasible	Small sample; benefits may overlap with PCI effects
[64]	Rodrigo et al.	2013	Intramyocardial Injection of Autologous Bone Marrow-Derived Ex Vivo Expanded Mesenchymal Stem Cells in Acute Myocardial Infarction Patients is Feasible and Safe up to 5 Years of Follow-up	Clinical	Bone Marrow	Intramyocardial Injection	A 5-year event-free survival comparable to controls; improved LV function at 12 months; safe and feasible	Small sample; nonrandomized control; underpowered to detect LV treatment effect
[65]	Haack-Sorensen et al.	2007	The influence of freezing and storage on the characteristics and functions of human mesenchymal stromal cells isolated for clinical use	Preclinical	Bone Marrow	-	Proliferation/differentiation capacities unchanged after freezing; comparable to fresh MSCs	MSC cultures are morphologically heterogeneous; no well-defined marker for BM-derived MSCs

## Data Availability

No new data were created in the creation of this article.
